# Highly Sensitive Uric Acid Detection Based on a Graphene Chemoresistor and Magnetic Beads

**DOI:** 10.3390/bios11090304

**Published:** 2021-08-29

**Authors:** Wangyang Zhang, Xiaoqiang Zhao, Lina Diao, Hao Li, Zhonghao Tong, Zhiqi Gu, Bin Miao, Zhan Xu, Han Zhang, Yue Wu, Jiadong Li

**Affiliations:** 1 Key Laboratory of Multifunctional Nanomaterials and Smart Systems, Suzhou Institute of Nano-Tech and Nano-Bionics, Chinese Academy of Sciences, Suzhou 215125, China; wyzhang2021@sinano.ac.cn (W.Z.); lndiao2020@sinano.ac.cn (L.D.); haoli2020@sinano.ac.cn (H.L.); zhtong2020@sinano.ac.cn (Z.T.); zqgu2017@sinano.ac.cn (Z.G.); bmiao2013@sinano.ac.cn (B.M.); zxu2020@sinano.ac.cn (Z.X.); 2 College of Mechatronic Engineering, North University of China, Taiyuan 030051, China; 15514414639@163.com; 3 School of Nano Technology and Nano Bionics, University of Science and Technology of China, Hefei 230026, China; 4 College of Mechatronic Engineering, Chengdu University of Technology, Chengdu 610059, China; 5 Electrical and Computer Engineering, Texas A&M University, College Station, TX 77843, USA; han.zhang@tamu.edu

**Keywords:** graphene, pH detection, uric acid detection, magnetic beads, chemoresistor

## Abstract

In this study, we developed a low-cost, reusable, and highly sensitive analytical platform for the detection of the human metabolite uric acid (UA). This novel analysis platform combines the graphene chemoresistor detection technique with a magnetic bead (MB) system. The heterojunction (single-layer graphene and HfO_2_ thin-film material) of our graphene-based biosensor worked as a transducer to detect the pH change caused by the specific catalytic reaction between UA and uricase, and hence acquires a UA concentration. Immobilization of uricase on MBs can decouple the functionalization steps from the sensor surface, which allows the sensor to be reusable. Our microsensor platform exhibits a relatively lower detection limit (1 μM), high sensitivity (5.6 mV/decade), a linear range (from 1 μM to 1000 μM), and excellent linearity (R^2^ = 0.9945). In addition, interference assay and repeatability tests were conducted, and the result suggests that our method is highly stable and not affected by common interfering substances (glucose and urea). The integration of this high-performance and compact biosensor device can create a point-of-care diagnosis system with reduced cost, test time, and reagent volume.

## 1. Introduction

The detection and quantification of biomarkers are essential for medical diagnostics, environmental monitoring, and bioresearch. Uric acid is a metabolite in whole blood and is crucial in the diagnosis of many clinical diseases [1], such as pre-eclampsia (PE) [2], gout [3], Parkinson’s disease (PD) [4], high blood pressure [5], and kidney disease [6]. Currently, the commonly used methodology for UA determination in clinical biofluids involves phosphotungstic acid colorimetry [7], UV absorbance [8], mass spectrometry (MS) [9], high-performance liquid chromatography, etc. [10]. Although some conventional methods provide high performance, they also exhibit some drawbacks, including complex operating procedures [11], relatively long time for sample preparation and detection, high cost due to the sophisticated instruments, and the need for professionals to detect [12]. Due to the aforementioned limitations of these conventional methods, there is an urgent need to develop low-cost, high-performance, and portable uric acid sensors for point-of-care detection [13].

Graphene-based nanomaterials are used as transducers of biosensors, which are involved in converting the interactions between the receptor and the target molecules into detectable measurements [14,15]. The graphene is exposed to enable functionalization of the graphene surface and binding of receptor molecules to the channel surface. The surface of the graphene channel is functionalized by binding receptors for the specific target of interest. Graphene biosensors exhibit the advantages of miniaturization, a low limit of detection, high sensitivity, and simplicity of design. Several research groups have reported the use of graphene sensors for the measurement and detection of chemical and biological components and target biomarkers for diseases, including cancer markers, DNA, and glucose [16,17]. However, the above-mentioned applications directly immobilize and functionalize receptor molecules on the graphene surface. In cases of multiple-target detection, the graphene surface of the sensor needs to be modified repeatedly and hence can severely affect the performance of the sensor. Especially, if the receptor binding is irreversible, sensor devices may not be reusable; as a result, the cost for each single detection can be significantly increased.

In the past decades, the combination of MBs with conventional biological assay techniques has achieved great success due to the excellent properties of MBs, which include scalable and reusable capabilities, a high specific surface area, less toxicity, powerful magnetism, and high loading of the sensitive matrix [18]. In the field of biosensing, intensive work on the application of magnetic beads in biosensors has been carried out, the majority of which employ magnetoresistive, Hall, thin-film transistor (TFT) nanoribbon [19], and surface plasmon resonance (SPR) sensors [20]. For example, biosensing based on MBs recently reported for clinical diagnosis of the inflammatory biomarker c-reactive protein (CRP) detects antibodies to disease vectors at clinical levels [21].

Here, we first proposed combining MBs and graphene chemoresistors as a microsensor platform for uric acid detection. This method overcomes the aforementioned drawbacks of the traditional sensor method. First, the proposed uric acid sensor is portable, reusable, and simple and has a low-cost fabrication process. Based on our calculation, the cost was approximately 10 USD. As it is reusable, the cost of each single test could be much lower. Second, the receptor functionalization step is achieved on the MB surface, breaking the main limitation of performance degradation caused by repeated modification of the graphene channel surface. Test results showed that the analysis platform exhibits excellent performance for the detection of uric acid. Miniaturization of the micro-biosensing platform endows itself with a promising prospect of application in a portable real-time instrument for point-of-care diagnostics.

## 2. Materials and Methods

### 2.1. Sensing Mechanism of Uric Acid Detection

**Figure 1:**
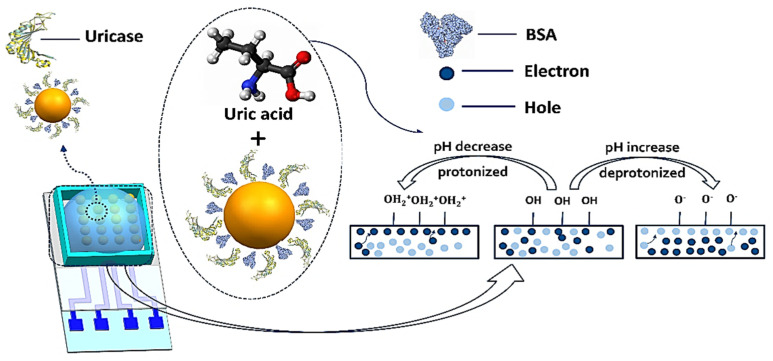
Working principle of the graphene-based biosensor for ultrasensitive detection of UA.

The mechanism of our graphene-based biosensor detection of uric acid is shown in Figure 1. Uric acid is converted to allantoin, hydrogen peroxide, and carbon dioxide during the catalytic reaction of uricase and leads to local pH shifts in the reaction channel. Hydroxyl groups on the surface of the channel can be protonated to be $\mathrm{OH}\begin{array}{c} +\\ 2 \end{array}$ as the pH decreases or deprotonated to be O^−^ as the pH increases [22]. Therefore, according to the configuration of the electrical double layer at the graphene/electrolyte interface, $\mathrm{OH}\begin{array}{c} +\\ 2 \end{array}$ make graphene n-doped and O^−^ make graphene p-doped; n-doped and p-doped graphene is able to modulate the channel conductance by doping holes or electrons, which is consistent with other graphene studies [23,24,25].

### 2.2. Materials

Iron chloride hexahydrate (FeCl_3_) and hydrochloric acid (HCl), which corrode copper foil, were purchased from Aladdin Reagent Co, Ltd. (Shanghai, China). Deionized water used in this work was purified using Milli-Q water purification. Buffer formulation ingredients, sodium sulfate (Na_2_SO_4_), sulfuric acid (H_2_SO_4_), sodium dihydrogen phosphate (NaH_2_PO_4_), sodium hydrogen phosphate (Na_2_HPO_4_), sodium bicarbonate (NaHCO_3_), and sodium carbonate (Na_2_CO_3_) included, were bought from Sinopharm Chemical Reagent Co, Ltd. (Shanghai, China). Dynabeads M-280 Tosylactivated (MBs), bovine serum albumin (BSA), and uricase, which was used to modify MBs, were purchased from Thermo Fisher Scientific. Phosphate-buffered saline (PBS) needed for this assay was purchased from Aladdin Reagent Co, Ltd. (Shanghai, China).

### 2.3. Equipment and Setup

An H-600 Hitachi scanning electron microscope (Tokyo, Japan) was used to characterize the scanning electron micrographs of the graphene morphology. Samples were imaged under the high-vacuum mode at magnifications of 800×. A Keysight 34465A digital multimeter was used to measure the source-drain voltage (*V_total_*) of the graphene chemoresistor. A Raman spectrometer (RamTracerTM-200 Raman Spectrometer) was performed to obtain Raman spectra to characterize the quality, layer number, and uniformity of graphene.

### 2.4. Biosensor Preparation

#### 2.4.1. Graphene Growth

The single-layer graphene films in our experiment were grown on copper foils by low-pressure CVD (LPCVD). First, copper substrates were pretreated by an annealing process, and the surface morphology of the copper substrate significantly improved. Then, copper foils were pre-cleaned in acetic acid at 35 °C for 10 min to remove the surface oxide [26]. Subsequently, methane (CH_4_) was introduced into the chamber with a flow rate of 35 sccm for 10 min to initiate the growth of graphene; at the same time, 35 sccm of hydrogen gas was added to the chamber (pressure of ~100 mTorr, temperature of ~1000 °C).

#### 2.4.2. Fabrication of the Graphene Biosensor

**Figure 2:**
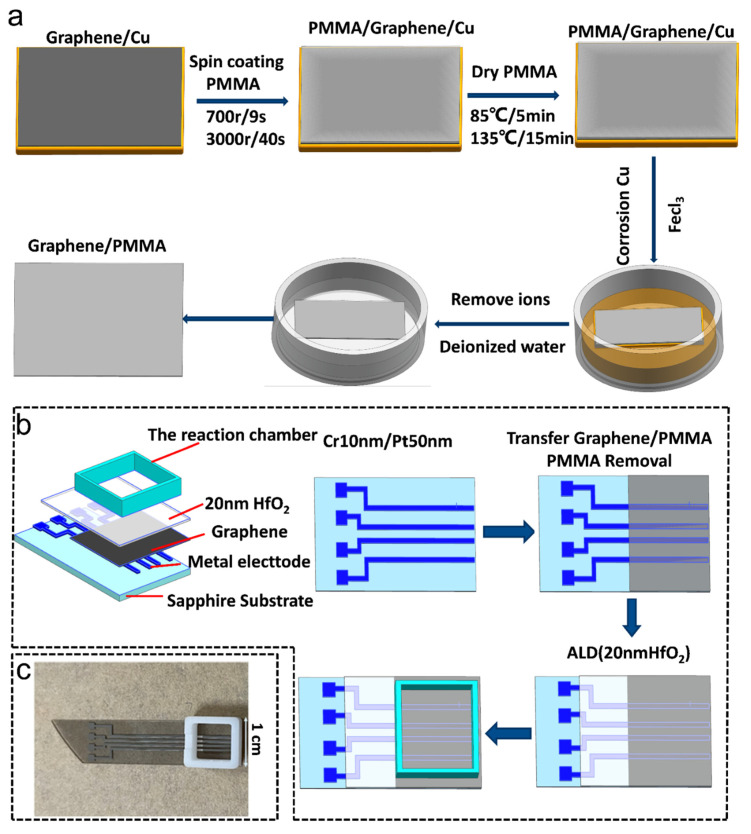
(**a**) Photograph of the sensor composed of a graphene chemoresistor with a reaction chamber. (**b**) Schematic diagram of etching monolayer graphene on Cu substrates. (**c**) Schematic illustration of the fabrication process of the graphene biosensor.

Figure 2a shows a schematic diagram of etching Cu substrates. Polymethyl methacrylate (PMMA) was spin-coated on top of graphene on a copper foil at 3000 rpm for 40 s. After coating, the sample was annealed at 135 °C for 15 min. To etch the copper foil, the PMMA-graphene-copper sample, with the copper side down, was placed in iron chloride hexahydrate solution (0.2 g/mL in deionized water) at room temperature. A PMMA/graphene block was immersed in dilute hydrochloric acid and deionized water to remove metal ions and bulk etchants. Figure 2c shows a schematic illustration of the manufacturing process of a graphene chemoresistor. In the first step, sapphire substrates were used after being cleaned with acetone and deionized water then we deposited electrodes by electron beam evaporation onto the substrate. In the second step, the obtained PMMA/graphene membrane was transferred to the Cr/Pt electrode substrate. The PMMA layer was removed by dipping in acetone for 30 min, and then the block was immersed in ethanol and isopropanol for 3 min each. Finally, HfO_2_ (20 nm) was deposited by atomic layer deposition (ALD).

#### 2.4.3. Magnetic Bead Functionalization with Uricase

**Figure 3:**
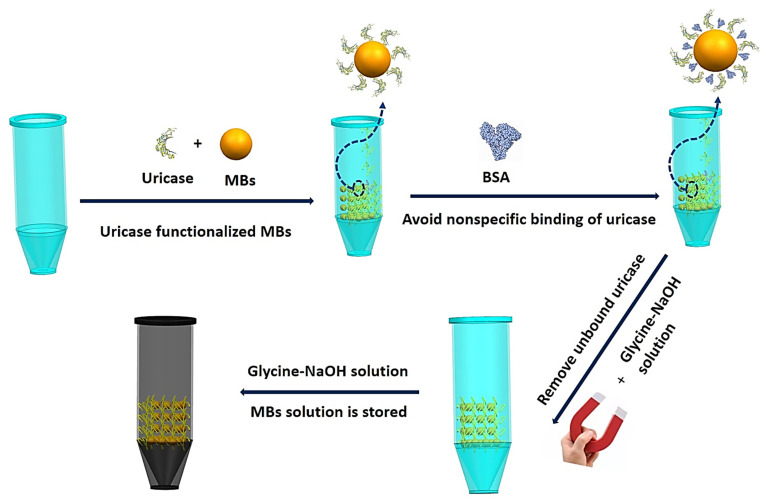
Schematic illustration of the MB modification process.

Figure 3 shows a schematic diagram of the MB modification process. Uricase (0.1 mg/mL in PBS) was mixed with the prepared beads in a tube for 2 h to conduct a modified reaction. The primary amine group of uricase can be covalently coupled with the p-toluene-sulfonyl group of the magnetic beads without any coupling agent. After that, the uricase-coated beads were blocked with bovine serum albumin (BSA, 20 μg/mL in PBS) solution to avoid nonspecific binding of uricase. The beads were collected using a hand-held magnet, and the unreacted uricase in glycine-NaOH buffer was rinsed repeatedly. Finally, functionalized magnetic beads were stored in glycine-NaOH buffer at 4 °C. When it was necessary to replace the modifying materials, we used a small hand-held magnet to clean them three times.

### 2.5. pH Value Measurement

To study the effect of pH on the performance of the graphene chemoresistor, acquisition of electrical signals was performed through a Keysight 34465A digital multimeter at a constant source and drain current (*I*) of 1 mA at different pH values of the solutions (from pH = 1.7 to pH = 4.1, from pH 6.0 to pH 8.0, and from pH 9.17 to pH 10.83). The pH change of the solutions was regulated by adjusting the proportion of buffer compositions. Respectively, neutral buffers of different pH values (from pH 6.0 to pH 8.0) were prepared by mixing 0.2 mol L^−1^ of NaH_2_PO_4_ and 0.2 mol L^−1^ of Na_2_HPO_4_, while acidic buffers of different pH values (from pH 1.7 to pH 4.1) were prepared by mixing 0.01 mol L^−1^ of Na_2_SO_4_ and 0.01 mol L^−1^ of H_2_SO_4_, and alkaline buffers of different pH values (from pH 9.17 to pH 10.83) were prepared by mixing 0.1 mol L^−1^ of Na_2_CO_3_ and 0.1 mol L^−1^ of NaHCO_3_.

### 2.6. Uric Acid Test Based on Beads

**Figure 4:**
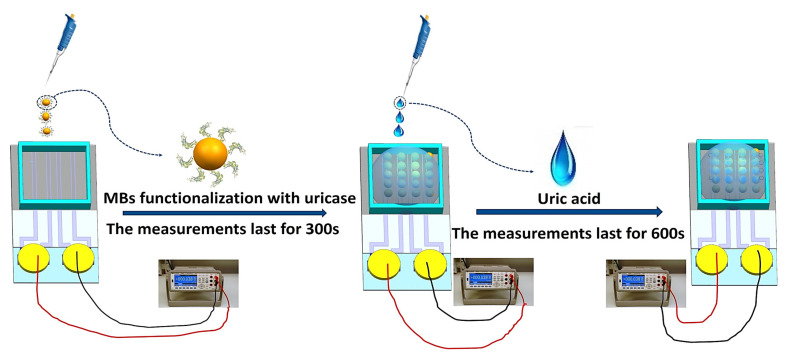
Schematic diagram showing the steps in the magnetic-bead-based detection of uric acid.

Figure 4 shows a schematic diagram of the steps in the magnetic-bead-based detection of uric acid. For measuring the response of different concentrations of uric acid, the change in the electrical signal was monitored using a computer-aided multimeter by applying a constant current of 1 mA [27]. First, MBs modified with uricase (40 μL, in glycine-NaOH solution at pH 8.5) were pipetted into the reaction chamber of the graphene sensor, and the signal remained stable with time. After 300 s, uric acid (20 μL, in glycine-NaOH solution at pH 8.5) at five different concentrations (0–1000 μM) was added and mixed (point UA on the graph), and the electrical signal was monitored (for typically 10 min).

The bead-based graphene biosensor was washed using deionized water when each test was finished, and the previously used MBs were cleaned out and refilled with new MBs and prepared for other sample detection. This biochemical analysis platform can not only detect the same UA in batches but also perform multiple analyses for different samples.

### 2.7. Studies of Interferences and Sensing Repeatability

Studies of interference were conducted. MBs modified with uricase were added to cover the reaction chamber with a micropipette. After 400 s, the electrical signal was stabilized, and 20 μL of urea and glucose solution of 1 mM concentration were added to the reaction chamber. Then, 20 μL of uric acid solution with a concentration of 1 mM was added, and the voltage change of the solution was measured using the sensor.

The sensing repeatability of the graphene-based biosensor was estimated through three consecutive measurements with the same sensor following the same procedure described above. In the pH repeatability value test, the pH change of the solution was regulated by adjusting the proportion of glycine-NaOH buffer compositions (from pH = 2 to pH = 11).

## 3. Results

### 3.1. Graphene Characterization

**Figure 5:**
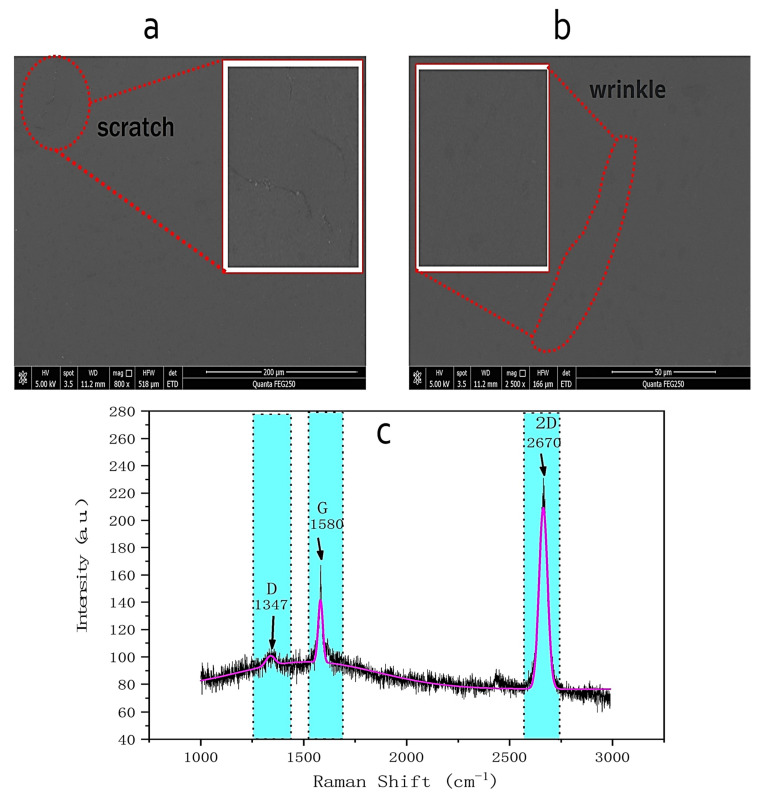
(**a**) SEM image of graphene on a transferred glass substrate (800× magnification). (**b**) SEM image of graphene on a transferred glass substrate (2500× magnification). (**c**) Raman spectra of graphene on a transferred sapphire substrate.

Scanning electron microscope (SEM) analysis was used to determine the morphology of graphene. SEM photomicrographs made at 800× magnification of graphene on a glass substrate are shown in Figure 5a. Graphene on a transferred sapphire substrate was a continuous film with a few scratches on the surface. These scratches formed during the transfer. SEM photomicrographs made at 2500× magnification of graphene on a sapphire substrate are shown in Figure 5b. Except for a small number of wrinkles, no resides were observed, which indicates that the graphene surface is clean. The quality of the graphene on a sapphire substrate was further measured by the Raman spectrum. As shown in Figure 5c, the three main peaks of graphene, such as D, G, and 2D peaks, were located near 1350 cm^−1^, 1590 cm^−1^, and 2700 cm^−1^, respectively. The related assignments of spectral peaks to chemical bonds can be found in our previously published works [28,29,30]. By comparing the intensities of G and 2D peaks, the *I*_2D_*/I*_G_ ratio was about 2, and more than 90% of the graphene film was determined as a single layer. In addition, the relatively weak D peak and the large 2D peak indicated the high quality and low defect density of graphene.

### 3.2. The Study of the Effects of pH

**Figure 6:**
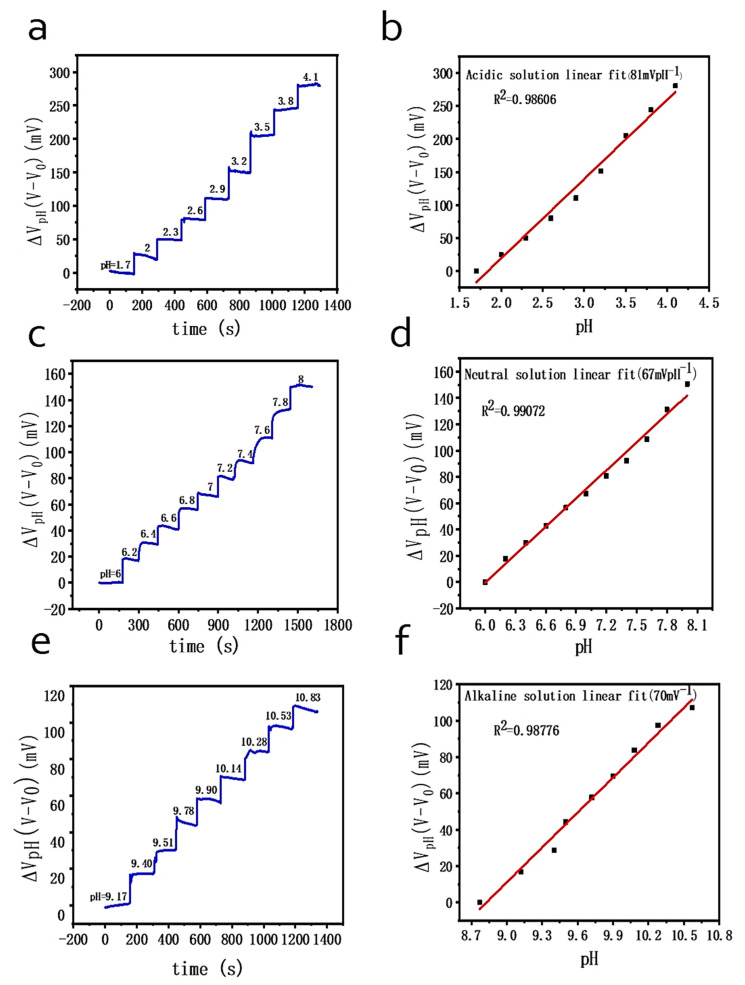
The voltage is a linear function of acidic solutions (**a**), neutral solutions (**c**), and alkaline solutions (**e**) with different pH values. The linearity for pH detection at pH = 1.7–4.1 (**b**), pH = 6–8 (**d**), and pH = 9.17–10.83 (**f**).

We evaluated the performance of our biosensor in acidic, neutral, and alkaline solutions (pH = 1.7–4.1, pH = 6–8, and pH = 9.17–10.83, respectively). Figure 6a,c,e clearly illustrates the significant changes in the source-to-drain voltage (*V_total_*). Measurements of the source-to-drain voltage (*V_total_*)as a function of time demonstrated stepwise increase along with the discrete change of the buffer’s pH. The results are shown in Figure 6b,d,f. It was observed that our graphene sensor possesses superb linearity for pH dependence characteristics. Besides, the sensitivities of our sensor was calculated as 67 mVpH^−1^ for the neutral solution, 81 mVpH^−1^ for the acidic solution, and 70 mVpH^-1^ for the alkaline solution. The standard deviations of the test were 2.29% (for pH = 6–8), 4.52% (for pH = 1.7–4.1), and 2.69% (for pH = 9.17–10.83).

To conclude, our biosensor exhibits good corresponding linearity and sensitivity performance in buffer solution, which suggests that our graphene chemoresistor is a microscale pH sensor. The testing results above also prove the feasibility using the biosensor to detect highly efficient reactions of uric acid and uricase.

### 3.3. Uric Acid Test Based on Beads

Uricase is immobilized on beads to specifically catalyze the oxidation of UA. Uricase hydrolyzes free uric acid according to Equation (1) below [31]:

(1) $$\mathrm{Uric acid}+H_{2}O+O_{2}\overset{\mathrm{Uricase}}{=}\mathrm{Allantoin}+H_{2}O_{2}+{\mathrm{CO}}_{2}$$

**Figure 7:**
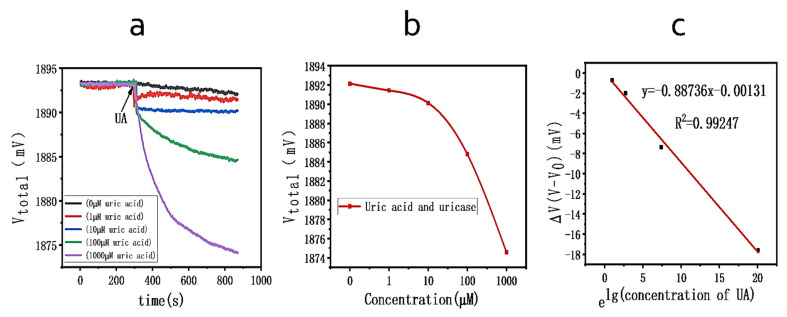
(**a**). Change in voltage for different concentrations of UA. (**b**) The plot of the final voltage (after 800 s) against uric acid concentration. (**c**) The linear relationship between measurement sensitivity and target UA concentrations.

As shown in Figure 7a, measurements indicated that the source–drain voltage (*V_total_*) of the graphene sensor decreased to varying degrees with the addition of different concentrations of uric acid. Figure 7b shows a similar standard curve extracted from the endpoint voltage (after 800 s) change. Experiments proved that a graphene chemoresistor based on magnetic beads can operate over a wide sensing range of uric acid concentrations (0 μM–1000 μM), which includes the clinically relevant range of uric acid in human serum (178 to 715 μM) [32]. In this study, the sensitivity of the sensor to detect UA was 5.6 mV/decade, and a good linear relationship (R^2^ = 0.995) was obtained when the UA concentration was changed from 0 μΜ to 1 mM, as shown in Figure 7c.

### 3.4. Studies of Interferences and Repeatability Test

**Figure 8:**
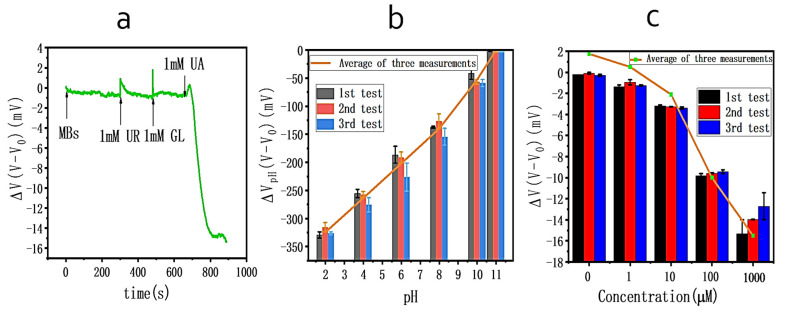
(**a**) Effect of potentially interfering substances on sensor response upon adding 1 mM glucose (GL) and 1 mM urea (UR) to 1 mM uric acid solution. (**b**) The three consecutive measurements of changes in pH at pH = 2–11. (**c**) Repeatability test of the UA sensor.

Selectivity is the ability of a biosensor to generate a positive result only from interactions. Figure 8a confirms the specificity of the uric acid sensor, and several common interfering substances in serum were used for interfering tests, including urea (UR) and glucose (GL) [33]. Adding UA did result in significant voltage changes. On the contrary, when urea and glucose were added, the average change in signal was 0.4 mV. The source-to-drain voltage slightly changed, which can be ignored. The results of the test prove that our biosensor is unaffected by interfering substances.

The graphene biosensor was then subjected to a repeatability test. Figure 8b,c shows that three independent tests of UA and pH were conducted. Before each test, the previously used MBs were cleaned out and refilled with new MBs. The resulting readouts of electric signals for most conditions (Figure 8b,c) only fluctuated at small ranges, and statistically, no significant differences were observed, except for the first and third tests at 1000 µM concentration (*p* < 0.01, N = 3). The above testing results demonstrated similar behaviors with voltage-time plots, which is similar to what was shown in Figure 6 and Figure 7, and also proved the acceptable reusability and stability of our biosensing system.

Compared with the traditional methods for UA detection, our system demonstrated better performance in terms of the detection range and limit of detection. The results are summarized in Table 1.

## 4. Conclusions

In summary, by atomic layer deposition, 20 nm of HfO_2_ was deposited on the surface of high-quality graphene to improve the pH response of the graphene sensor. After verifying the feasibility of the graphene biosensor to detect the change in pH in acidic and alkaline environments, it was combined with magnetic beads for the detection of uric acid. The low-cost and scalable analysis platform exhibited excellent performance for detecting uric acid, including a lower detection limit (1 μM), good sensitivity (5.6/dec), and excellent linearity (0.995). The sensor showed good real-time detection performance, good stability, simple operation, and specificity for uric acid. Furthermore, this proposed method can potentially be applied to biological sample detection in whole blood and serum in the future after optimization. All of these features suggest that our platform has the potential to be a competitive method in the field of healthcare monitoring and clinical diagnosis.

## Figures and Tables

**Table 1:** Comparison of the state-of-the-art analytical performance of different biosensors for the detection of UA.

Method	Linear Range (mM)	Sensitivity (mV·dec^−1^·cm^−2^)	Ref.
MPx-11/Au	0.005–0.15	NR	[34]
ZnO nanowires/Au	0.001–1	29 ± 5	[35]
Sm_2_TiO_5_/Si	0.01–0.04	18.6	[36]
Trp-GR/GCE	0.01–1	NR	[34]
ZnO nanoflakes/glass	0.0005–1.5	66 ± 8	[35]
MWCNT-NH2/AuNPs	0.001–0.2	NR	[37]
KCM	0.004–0.8	NR	[38]
Graphene/HfO_2_	0.001–1	89.6	This work

## Data Availability

The data presented in this study are available on request from the corresponding author.

## References

[1] Chen J.C., Chung H.H., Hsu C.T., Tsai D.M., Kumar A.S., Zen J.M. (2005). A disposable single-use electrochemical sensor for the detection of uric acid in human whole blood. Sens. Actuators B Chem..

[2] Poon L.C., Nicolaides K.H. (2014). Early prediction of preeclampsia. Obstet. Gynecol. Int..

[3] Seegmiller J.E., Grayzel A.I., Laster L. (1961). Uric acid production in gout. J. Clin. Investig..

[4] Schlesinger I., Schlesinger N. (2008). Uric acid in Parkinson’s disease. Mov. Disord. Off. J. Mov. Disord. Soc..

[5] Sundstrom J., Sullivan L., D’Agostino R.B., Levy D., Kannel W.B., Vasan R.S. (2005). Relations of serum uric acid to longitudinal blood pressure tracking and hypertension incidence. Hypertension.

[6] Chonchol M., Shlipak M.G., Katz M.R., Sarnak M.J., Anne B., Siscovick D.S., Kestenbaum B., Carney J.K., Fried L.F. (2007). Relationship of uric acid with progression of kidney disease. Am. J. Kidney Dis..

[7] De Almeida F.L., dos Santos Filho S.G. (2019). Nitrite Detection in Near-Neutral-pH Electrolytes by Differential Pulse Voltammetry under Cross Interference of Uric Acid, Ascorbic Acid, and Paracetamol. IEEE Sens. Lett..

[8] Kanďár R., Drábková P., Hampl R. (2011). The determination of ascorbic acid and uric acid in human seminal plasma using an HPLC with UV detection. J. Chromatogr. B.

[9] Kim K.M., Henderson G.N., Ouyang X.S., Frye R.F., Sautin Y.Y., Feig D.L. (2009). A sensitive and specific liquid chromatography-tandem mass spectrometry method for the determination of intracellular and extracellular uric acid. J. Chromatogr. B.

[10] Dai X.H., Fang X., Zhang C.M., Xu R.F., Xu B. (2007). Determination of serum uric acid using high-performance liquid chromatography (HPLC)/isotope dilution mass spectrometry (ID-MS) as a candidate reference method. J. Chromatogr. B.

[11] Kuo P.Y., Chen Y.Y. (2021). A Novel Low Unity-Gain Frequency and Low Power Consumption Instrumentation Amplifier Design for RuO₂ Uric Acid Biosensor Measurement. IEEE Trans. Instrum. Meas..

[12] Sheng Z.H., Zheng X.Q., Xu J.Y., Bao W.J., Wang F.B., Xia X.H. (2012). Electrochemical sensor based on nitrogen doped graphene: Simultaneous determination of ascorbic acid, dopamine and uric acid. Biosens. Bioelectron..

[13] Yang Y., Song Y., Bo X., Min J., Pak O.S., Zhu L., Wang M., Tu J., Kogan A., Zhang H. (2020). A laser-engraved wearable sensor for sensitive detection of uric acid and tyrosine in sweat. Nat. Biotechnol..

[14] Wang J.Q., Liu G., Leung K.C.-F., Romaric L., Xuan L.P., Xiang J., Wang Y. (2015). Opportunities and challenges of fluorescent carbon dots in translational optical imaging. Curr. Pharm. Des..

[15] Welch E.C., Powell J.M., Clevinger T.B., Fairman A.E., Shukla A. (2021). Advances in Biosensors and Diagnostic Technologies Using Nanostructures and Nanomaterials. Adv. Funct. Mater..

[16] Wu D., Yu Y., Jin D., Xiao M.M., Zhang Z.Y., Zhang G.J. (2020). Dual-aptamer modified graphene field-effect transistor nanosensor for label-free and specific detection of hepatocellular carcinoma-derived microvesicles. Anal. Chem..

[17] Dontschuk N., Stacey A., Tadich A. (2015). A graphene field-effect transistor as a molecule-specific probe of DNA nucleobases. Nat. Commun..

[18] Geng P., Zhang X., Teng Y., Fu Y., Xu L., Xu M., Jin L., Zhang W. (2011). A DNA sequence-specific electrochemical biosensor based on alginic acid-coated cobalt magnetic beads for the detection of *E. coli*. Biosens. Bioelectron..

[19] Kim K.T., Jung J.W., Jo W.H. (2013). Synthesis of graphene nanoribbons with various widths and its application to thin-film transistor. Carbon.

[20] Homola J., Yee S.S., Gauglitz G. (1999). Surface plasmon resonance sensors. Sens. Actuators B Chem..

[21] Hu C.X., Zeimpekis L., Sun K., Andersont S., Ashburn P., Morgan H. (2016). Low-cost nanoribbon sensors for protein analysis in human serum using a miniature bead-based enzyme-linked immunosorbent assay. Anal. Chem..

[22] Li C.Y., Ma F.X., Wu Z.Q., Gao H.L., Shao W.T., Wang K., Xia X.H. (2013). Solution-pH-modulated rectification of ionic current in highly ordered nanochannel arrays patterned with chemical functional groups at designed positions. Adv. Funct. Mater..

[23] Wangyang F., Cornelia N., Oren K., Alexey T., Markus W., Michel C., Christian S. (2011). Graphene transistors are insensitive to pH changes in solution. Nano Lett..

[24] Lei N., Li P., Xue W., Xu J. (2011). Simple graphene chemiresistors as pH sensors: Fabrication and characterization. Meas. Sci. Technol..

[25] Fowler J.D., Allen M.J., Tung V.C., Yang Y., Kaner R.B., Weiller B.H. (2009). Practical chemical sensors from chemically derived graphene. ACS Nano.

[26] Bae S., Kim H., Lee Y. (2010). Roll-to-roll production of 30-inch graphene films for transparent electrodes. Nat. Nanotechnol..

[27] Morales M.A., Halpern J.M. (2018). Guide to selecting a biorecognition element for biosensors. Bioconjugate Chem..

[28] Zhang H., Silva A.C., Zhang W., Rutigliano H., Zhou A. (2020). Raman Spectroscopy characterization extracellular vesicles from bovine placenta and peripheral blood mononuclear cells. PLoS ONE.

[29] Zhang H., Zhang W., Xiao L.F., Liu Y., Gilbertson T.A., Zhou A.H. (2019). Use of surface-enhanced Raman scattering (SERS) probes to detect fatty acid receptor activity in a microfluidic device. Sensors.

[30] Zhang H., Xiao L., Li Q., Qi X., Zhou A. (2018). Microfluidic chip for non-invasive analysis of tumor cells interaction with anti-cancer drug doxorubicin by AFM and Raman spectroscopy. Biomicrofluidics.

[31] Zhao Y., Yang X., Lu W. (2009). Uricase based methods for determination of uric acid in serum. Microchim. Acta.

[32] Behera S., Raj C.R. (2007). Mercaptoethylpyrazine promoted electrochemistry of redox protein and amperometric biosensing of uric acid. Biosens. Bioelectron..

[33] Ali S.M.U., Alvi N.H., Ibupoto Z., Willander M., Danielsson B. (2011). Selective potentiometric determination of uric acid with uricase immobilized on ZnO nanowires. Sens. Actuators B Chem..

[34] Li L., Wang Y., Pan L., Shi Y., Cheng W., Shi Y., Yu G. (2015). A nanostructured conductive hydrogels-based biosensor platform for human metabolite detection. Nano Lett..

[35] Ali S.M.U., Ibupoto Z.H., Kashif M., Hashim U., Willander M. (2012). A potentiometric indirect uric acid sensor based on ZnO nanoflakes and immobilized uricase. Sensors.

[36] Wu M.H., Lin T.W., Huang M.D. (2010). Label-free detection of serum uric acid using novel high-k Sm2TiO5 membrane-based electrolyte-insulator-semiconductor. Sens. Actuators B Chem..

[37] Guan Q.G., Guo H., Xue R., Wang M.Y., Zhao X., Fan T., Yang W., Xu M., Yang W. (2021). Electrochemical sensor based on covalent organic frameworks-MWCNT-NH2/AuNPs for simultaneous detection of dopamine and uric acid. J. Electroanal. Chem..

[38] Kim I., Kim Y.I., Lee S.W., Jung H.G., Lee G.L., Yoona D.S. (2021). Highly Permselective Uric Acid Detection Using Kidney Cell Membrane—Functionalized Enzymatic Biosensors. Biosens. Bioelectron..

